# Supplementary figures and images for: Advancing the design of the kissing bug kill trap for surveillance of triatomines
Source: PLoS Negl Trop Dis. 2026 Feb 27;20(2):e0014005. doi: 10.1371/journal.pntd.0014005 (PMC12948065; doi:10.1371/journal.pntd.0014005)

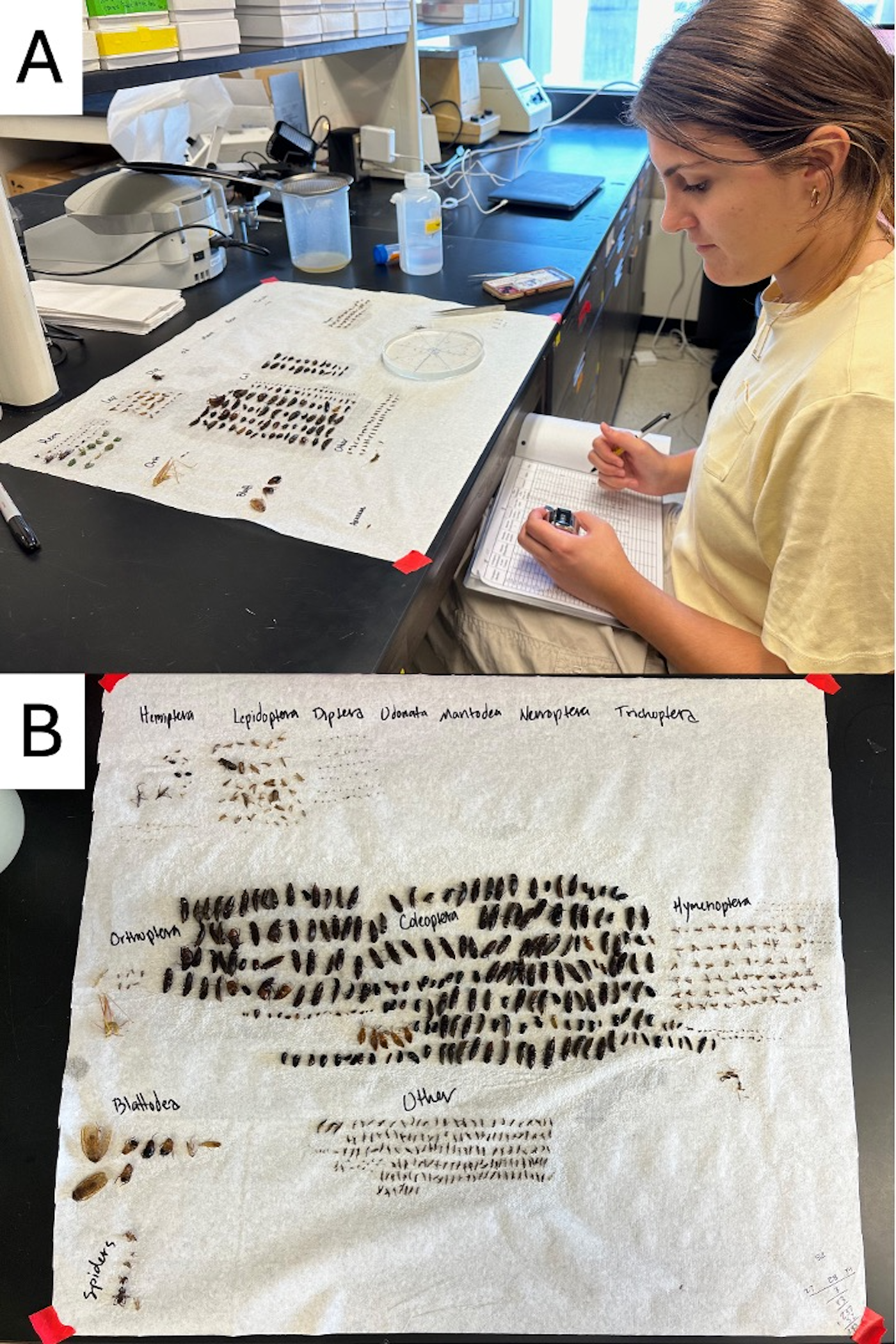

Supplement: S1 Fig — Triatomines were removed and counted as the first step as they are larger than most of the by-catch and have a distinctive shape. (TIFF) [file pntd.0014005.s003.tiff]

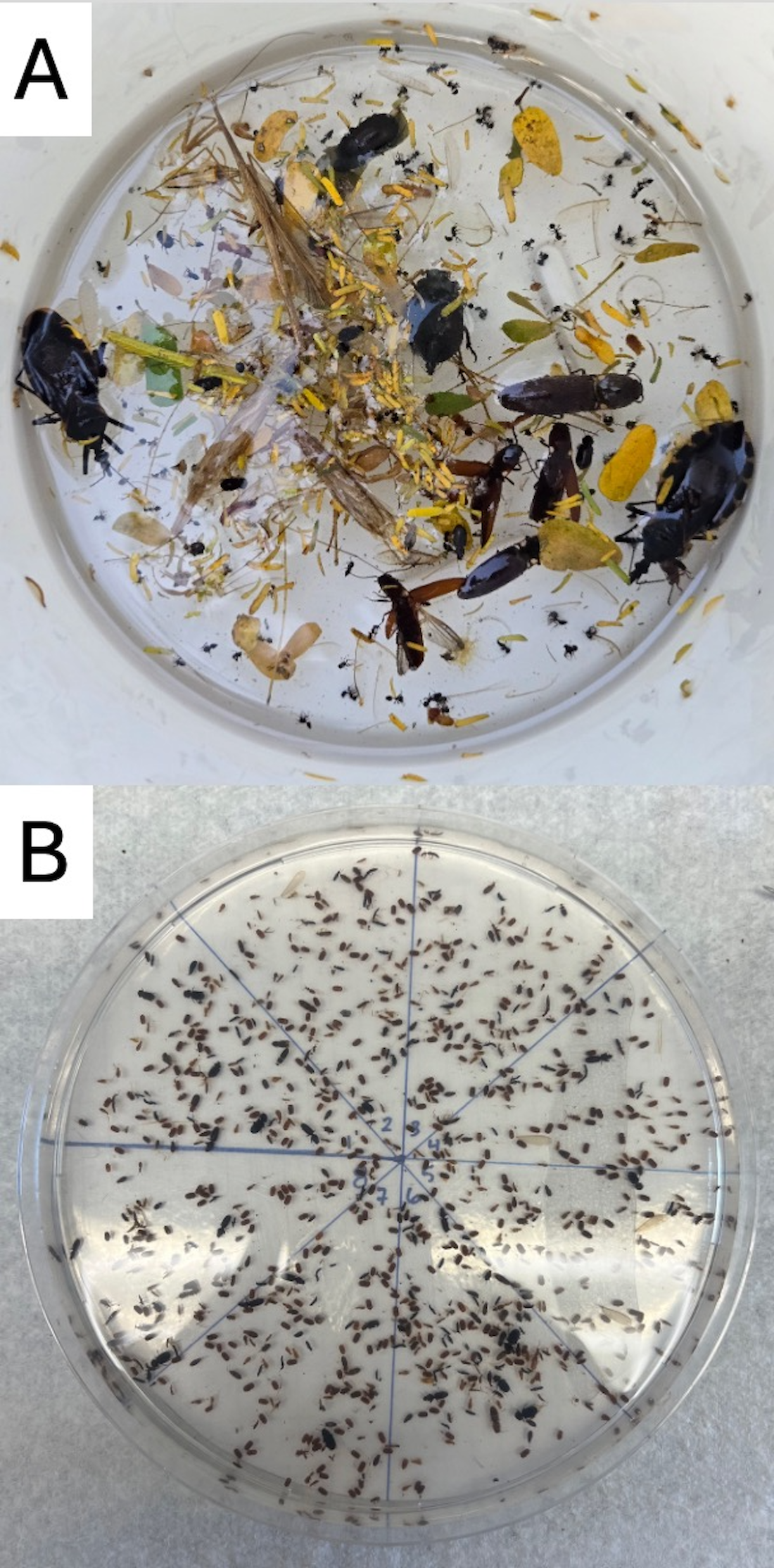

Supplement: S2 Fig — (TIFF) [file pntd.0014005.s004.tiff]
